# State of the art of mobile health technologies use in clinical arrhythmia care

**DOI:** 10.1038/s43856-024-00618-4

**Published:** 2024-10-29

**Authors:** Arun R. Sridhar, Jim W. Cheung, Rachel Lampert, Jennifer N. A. Silva, Rakesh Gopinathannair, Juan C. Sotomonte, Khaldoun Tarakji, Mark Fellman, Jonathan Chrispin, Niraj Varma, Rajesh Kabra, Nishaki Mehta, Sana M Al-Khatib, Jacob J. Mayfield, Rachita Navara, Bharath Rajagopalan, Rod Passman, Yann Fleureau, Maully J Shah, Mintu Turakhia, Dhanunjaya Lakkireddy

**Affiliations:** 1 Cardiac Electrophysiology, Pulse Heart Institute, Multicare Health System, Tacoma, Washington USA; 2 Division of Cardiology, Department of Medicine, Weill Cornell Medicine, New York, NY USA; 3 Cardiovascular Medicine, Yale School of Medicine, New Haven, CT USA; 4 Washington University School of Medicine/St. Louis Children’s Hospital, St. Louis, MO USA; 5 Kansas City Heart Rhythm Institute, Overland Park, KS USA; 6 Cardiovascular Center of Puerto Rico/University of Puerto Rico, San Juan, PR USA; 7 Medtronic Inc, Minneapolis, MN USA; 8 Fellman Device Group LLC, Rockville, MD USA; 9 Division of Cardiology, Johns Hopkins University, Baltimore, MD USA; 10 Heart and Vascular Institute, Cleveland Clinic, Cleveland, OH USA; 11 William Beaumont Oakland University School of Medicine, Rochester, MI USA; 12 Division of Cardiology, Duke University Medical Center, Durham, England; 13 Presbyterian Heart Group, University of New Mexico School of Medicine, Albuquerque, New Mexico, USA; 14 Division of Cardiology, University of California at San Francisco, San Francisco, CA USA; 15 Prairie Heart Institute, Springfield, IL USA; 16 Division of Cardiology, Northwestern University School of Medicine, Chicago, IL USA; 17 Cardiologs, Inc, Paris, France; 18 Division of Cardiology, The Children’s Hospital of Philadelphia, Philadelphia, PA USA; 19 Center for Digital Health, Stanford University Stanford, Stanford, CA USA

**Keywords:** Cardiology, Cardiac device therapy

## Abstract

The rapid growth in consumer-facing mobile and sensor technologies has created tremendous opportunities for patient-driven personalized health management. The diagnosis and management of cardiac arrhythmias are particularly well suited to benefit from these easily accessible consumer health technologies. In particular, smartphone-based and wrist-worn wearable electrocardiogram (ECG) and photoplethysmography (PPG) technology can facilitate relatively inexpensive, long-term rhythm monitoring. Here we review the practical utility of the currently available and emerging mobile health technologies relevant to cardiac arrhythmia care. We discuss the applications of these tools, which vary with respect to diagnostic performance, target populations, and indications. We also highlight that requirements for successful integration into clinical practice require adaptations to regulatory approval, data management, electronic medical record integration, quality oversight, and efforts to minimize the additional burden to health care professionals.

## Abstract (web summary)

Sridhar et al. review the opportunities that mobile health technologies offer to improve the care of patients with cardiac arrhythmias. The current status, challenges, and future opportunities to use these technologies to predict, screen, diagnose, and manage of cardiac arrhythmias are highlighted.

## Introduction

The rapid growth in mobile health (mHealth) technologies has created tremendous opportunities for consumer-driven health management. Current generations of smartphones and other consumer mobile devices incorporate sensors and components that are either developed for the purpose of health monitoring, such as photoplethysmography (PPG), activity sensors, hand-held ECG systems, or re-purposed for health and activity monitoring, such as cameras, a global positioning system (GPS), accelerometers, gyroscopes, microphones, or a digital compass. Increased affordability of these mobile devices, coupled with improved high-speed internet access, has created an opportunity to democratize healthcare access for patients. Cardiac arrhythmia care is particularly well-suited to benefit from these burgeoning accessible consumer health technologies. These technologies allow for real-time monitoring and early detection of arrhythmias, enabling timely medical intervention and empowering patients to manage their conditions more effectively.

Cardiac arrhythmias encompass a range of conditions where the heart beats irregularly, too quickly, or too slowly, disrupting its ability to pump blood effectively. Common types include atrial fibrillation (AF), characterized by chaotic atrial beats that increase stroke risk; ventricular tachycardia (VT) and ventricular fibrillation (VF), both serious conditions where rapid beats from the ventricles can lead to cardiac arrest; and bradycardia, where slow heart rates cause fatigue and dizziness. Supraventricular tachycardia (SVT) involves rapid heart rates originating above the ventricles, while premature atrial contractions (PACs) and premature ventricular contractions (PVCs) are extra beats disrupting regular rhythm. While a comprehensive review of cardiac arrhythmias is beyond the scope of this article, readers can refer to several excellent reviews available on this topic^1^.

In this review, we outline the practical utility of the available and emerging mHealth technologies related to cardiac arrhythmia care. We review available literature to summarize what is known about the use of these technologies for the prediction, screening, diagnosis, and management of cardiac arrhythmias. We review the benefits, limitations, and potential risks of this digital technological revolution in arrhythmia care; and identify the gaps in knowledge and future directions. Box 1 provides a framework of definitions for digital health, mHealth, and digital medicine to clarify these often interchangeably used terms, and familiarize readers with accepted concepts in healthcare.

Box 1: Definitions While no standardized definitions exist, the terms Digital health, Digital Medicine, and mHealth are often used in healthcare interchangeably. Here, we provide a framework of definitions to familiarize the reader with some accepted concepts.

Digital health—a broad term that refers to the use of information and communication technologies to enhance healthcare delivery in a more personalized and precise fashion^1^. Digital health includes the use of both hardware and software solutions to achieve this goal through smart devices, advanced computational analysis such as machine learning (ML) and artificial intelligence (AI), telehealth, health information technology (HIT), and data science.

mHealth—a component of digital health. It refers to using wireless mobile-based solutions and ‘smart’ internet-connected consumer devices to deliver healthcare. It can include various hardware solutions such as health monitoring sensors (PPG, ECG, and accelerometer) or software solutions such as healthcare apps.

Digital medicine—the practice of evidence-based medicine using digital health tools and providing health promotion, preventative health, diagnosis, and management of health conditions across individuals and populations^2^. Specifically, the discerning features of digital medicine are regulatory oversight and evidence-based practice; as compared to digital health, which is a much broader term with more liberal parameters and includes lifestyle and fitness products, which do not require stringent regulatory oversight.

## Currently available mobile heart monitoring technologies

**Fig. 1:**
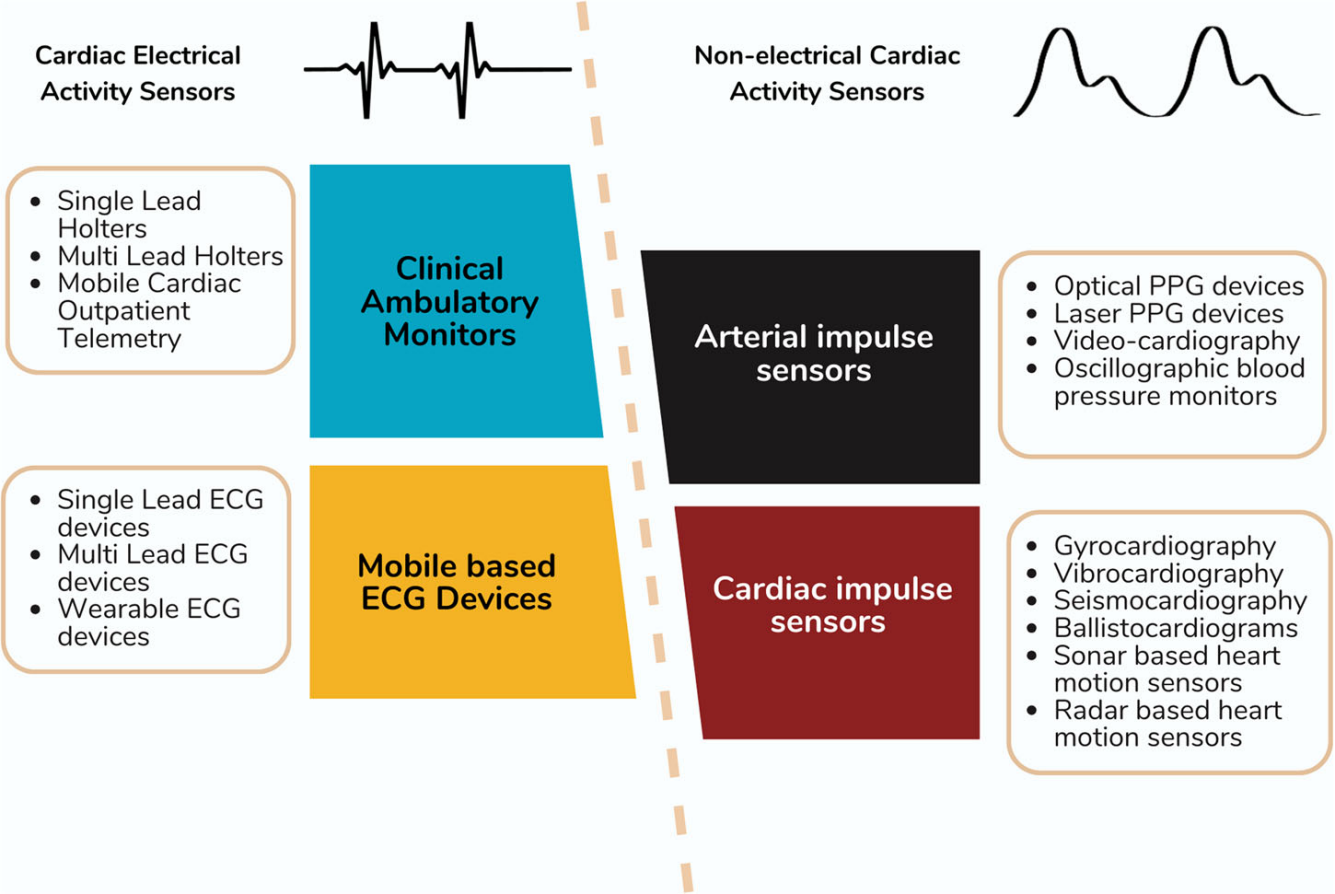
Cardiac rate and rhythm monitoring sensor technologies. We can broadly divide the cardiac monitoring technologies into electrical activity sensors which include electrocardiograms, and non-electrical cardiac activity sensors which essentially sense the mechanical impulse generated by the heart either centrally or peripherally. ECG electrocardiogram, PPG photoplethysmography.

Cardiac monitoring technologies can be broadly divided into cardiac electrical activity sensors which include electrocardiograms, and non-electrical cardiac activity sensors which sense the impact of the mechanical pulse generated by the heart either centrally in the heart itself, or peripherally, within arteries (Fig. 1).

### Cardiac electrical activity sensors

Systems have been available for ambulatory ECG recordings since the 1980s. Ambulatory ECG monitors are portable devices that patients wear to record the electrical activity of their heart while they go about their daily activities, allowing for the detection and diagnosis of arrhythmias outside of a hospital setting. These ambulatory ECG monitors can be used continuously for both short-term and long-term cardiac diagnostics and can monitor multiple signals simultaneously by using multi-channel recording^2^. There are three main components: wearable sensors for collecting parameters of interest; a communications interface (e.g., home monitor or smartphone); and an analytics platform that analyzes the incoming information. The analytics can be processed retroactively or in real time with notifications generated and communicated. The recording is automatic, with pre-set parameters and the option for patient-activated or triggered recording^3^.

Ambulatory ECG devices can be broadly divided into traditional clinician-driven ambulatory monitors and mobile consumer-driven ECG technologies. Clinical ambulatory monitors typically use wet ECG electrodes (gel interface to the skin); while mobile ECG technologies use dry electrode interface (Table 1). These devices have undergone significant advances in the last decade. Patch-form ambulatory ECG devices have supplanted multi-lead monitors for many clinical uses due to reduced size, leadless design, and water-resistant capabilities^3,4^. Recently, multi-lead patch ECG devices have become available which claim better discrimination of arrhythmias, as well as cardiac localization^5^. Smartphone-based mobile ECG devices on the market include devices that range from one lead to twelve leads and enable patients to monitor their rhythms and share recordings with providers. These devices generally pair with smartphones via Bluetooth and are analyzed either locally with in-app software or via a programming interface that communicates with algorithms in the internet cloud to provide an ambulatory diagnosis of cardiac events^4^.

**Table 1:** Differences between clinician-driven cardiac ambulatory ECG monitors and consumer-driven ECG monitoring technologies

Clinician-driven ambulatory ECG monitors	Consumer-driven mobile ECG monitors
Gel electrode-skin interface (less prone to movement artifact)	Dry electrode-skin interface (dry electrodes have significant movement artifact potential which is reduced by post-processing techniques on the mobile platform)
Continuous but finite duration contact with the subject’s body	Intermittent consumer-directed contact but not typically limited in total duration of use
Data downloaded and processed in the healthcare facility	Data processing within the ECG machine and or an IOT-connected smart mobile device
Typically, it does not have a mobile platform	Mobile platform
Higher cost and limited access	Low-cost and more accessible
Regulatory and payment models are well-established	Regulatory and payment models are evolving

### Non-electrical cardiac activity sensors

#### PPG-based monitors

Pulse oximeters commonly use PPG to detect sudden changes in the blood volume using light reflected or transmitted through the skin^6^. Smartphone flash and camera-based PPG applications have been developed that can measure heart and respiratory rates and can be incorporated into wristwatches or chest straps. Facial PPG is a technique that uses the camera of a smartphone or other device to detect subtle changes in the skin color of the face caused by the pulsatile blood flow under the skin. Facial PPG has also been demonstrated to be successful in detecting AF^7,8^.

#### Oscillometric blood pressure monitors

Automatic oscillometric blood pressure monitors can detect fluctuation in pulse beat intervals and apply specific algorithms to detect irregular heartbeat. While some of these algorithms have not been specifically developed to detect irregular heartbeat, others are specific for AF detection^9,10^. These devices have high specificity and sensitivity compared to manual pulse palpation for AF detection, enabling more effective AF screening^11^.

#### Ballistocardiography (BCG)

BCG measures the whole-body recoil due to cardiac activity, usually through sensors placed on surfaces that support the body. It is therefore another tool that can diagnose cardiovascular disorders, as well as sleep-disordered breathing^12,13^. Currently, available BCG sensors can be embedded unobtrusively in pillows, mattresses, chairs, beds, and weighing scales and can monitor multiple parameters including heart rate, respiration, snoring, coughing, and movement. In addition to the detection of arrhythmia, they may also help diagnose sleep apnea, a risk factor for AF^12^.

#### Sound and vibration-based cardiac impulse detection systems

Seismocardiography (SCG) is a technique that records chest wall sounds and vibrations occurring due to cardiac mechanics. This method can be embedded into soft wearable systems, such as T-shirts, allowing for continuous and non-intrusive monitoring of heart function^14,15^. Triaxial accelerometers, which detect small motion changes based on inertia, enable cardiac monitoring even when the device, like a smartphone, is in a bag or pocket. Complementing this, gyrocardiography measures angular motion and captures precordial microvibrations, providing detailed beat-to-beat hemodynamic monitoring^16^. These technologies have been integrated into various wearable devices such as glasses, patches, wristbands, headbands, textile shirts, and necklaces for minimally intrusive cardiac monitoring. Recently, smart speakers have also been studied to detect R-R intervals by algorithmically converting them into short-range sonars. This approach can identify arrhythmias in individuals in close proximity to the device^17^. Additionally, using vocal feature analysis for detecting AF has been shown to be feasible and accurate^18^.

### Non-cardiac sensors which are helpful in cardiac rhythm care

Because many of the sensor methodologies discussed above measure vibrations or movement, they can also be used to measure other health-related parameters. Activity sensors, incorporated in smartwatches, wearable bands, implantable cardiac monitors or devices, and pedometers measure movements and convert them to estimates of physical activities^19^. Because these data track activity objectively they can improve knowledge of how a person’s lifestyle could be impacting their arrhythmia, and hence be managed to improve health. Increased physical activity has not only been associated with decreased risk of AF in the general population, but also used to manage co-morbidities including hypertension, obesity, diabetes mellitus (DM), heart failure (HF), and coronary artery disease^20^.

Wearable devices are now available that monitor sleep that are minimally intrusive, or do not require wearable sensors^21^. For example, sleep sensing technology developed by Google uses low-energy radar to detect movement and breathing during sleep^22^. Additional sensors in the detecting device monitor for snoring and coughing. Detecting snoring can help identify sleep apnea, which often goes undiagnosed but is a risk factor for cardiac arrhythmias^21^. Cough monitoring can provide insights into respiratory issues that might affect heart function, especially in patients with HF or other cardiac conditions^23^.

Diabetes is a common comorbidity in cardiac conditions, and its management is essential for improving outcomes in conditions such as AF and other arrhythmias. There has been considerable interest in non-invasive continuous monitoring of blood glucose. Several ongoing studies are using ML models and feature extraction techniques to enable ECG-based continuous glucose monitoring^24^. These can potentially expand the role of wearable devices with ECG sensors for hyperglycemia detection in a non-invasive manner.

## mHealth applications for arrhythmia care

Arrhythmias including AF, PAC, PVC, SVT, and VT are usually paroxysmal (suddenly increase or recur). The critical step of establishing symptom-rhythm correlation has required the use of medical-grade ambulatory electrocardiogram (ECG) monitors, 24–48 Holter, or longer-term event recorders. These tools have been limited in the time they can monitor a patient, which is problematic if no symptoms are experienced while wearing the monitor. mHealth devices, particularly smartphone-based ECG and PPG technology, facilitate relatively inexpensive, long-term rhythm monitoring. This can enable the patient themselves to detect arrhythmias, albeit with some limitations. In this section, we discuss the evidence that these devices can be used to screen for arrhythmias, confirm diagnosis, and assess response to treatment, including medical or interventional procedures, such as catheter ablation.

### AF screening

Direct-to-consumer technologies such as handheld or wearable devices, and smartphone apps have provided new screening tools for AF^8,25,26^. Clinical use of these screening options requires them to be clinically validated, as well as proof that they do improve outcomes in the general population if used more widely than for targeted risk factor-based screening^27^.

The SAFE study showed that pulse palpation in older patients in a routine or opportunistic fashion increased overall AF detection^28^. However, this study did not have a control group. The STROKESTOP study targeted people in Sweden who were 75–76 years old. Nearly 29,000 individuals were randomly assigned to screening versus control groups, with the former using a handheld ECG transmitter to record intermittent ECGs for 14 days^29^. Notably, 51.3% of people who were invited underwent screening, of whom 3% were diagnosed with AF, and subsequently started on anticoagulation. The combined endpoint was a composite of ischemic or hemorrhagic stroke, systemic embolism, bleeding leading to hospitalization, and all-cause death. The screening led to a small but significant reduction in the combined endpoint of ischemic or hemorrhagic stroke, systemic embolism, bleeding leading to hospitalization, and all-cause death.

Several studies using different types of smartwatches have been conducted. The Apple Heart, Huawei Heart, and Fitbit studies employed smartwatch PPG technology for the detection of irregular pulses, followed by the use of ECG patch monitoring to confirm any suspected AF diagnoses^30–32^.

In the Apple Heart Study, which encompassed nearly 420,000 participants, an irregular pulse notification rate of 0.5% was observed, with 34% of these individuals with irregular pulse notifications ultimately being diagnosed with AF through ECG. Similarly, the Huawei Heart Study and the Fitbit study demonstrated large-scale smartwatches enabled monitoring and detection of AF. However, all these studies lacked randomization and conventional control groups as part of their study design.

In the eBRAVE-AF randomized cross-over clinical trial, over 5500 individuals without AF were randomly assigned to digital screening using a smartphone app or usual care. A certified app was used for digital screening to monitor pulse waves, with abnormal results confirmed by external ECG loop recorders. The primary goal was to identify newly diagnosed AF within 6 months and treat it with oral anticoagulation. The trial found that digital screening more than doubled the detection rate of treatment-relevant AF compared to usual care, with odds ratios of 2.12 and 2.75 in the two phases of the trial^33^.

The ongoing HEARTLINE trial (a heart health study using digital technology to investigate if early AF diagnosis reduces the risk of thromboembolic events like stroke in the real-world environment) is a randomized controlled research study that aims to assess if accessible technologies such as Apple Watch with a heart health engagement program, can help with early detection of AF and potentially improve clinical outcomes^34^. The LOOP trial evaluated whether AF screening-based anticoagulant use can prevent stroke in high-risk individuals. A total of 6004 patients (25% implantable loop recorder (ILR) monitoring and 75% usual care) were followed for 64.5 months. AF was diagnosed in 31% of the ILR group and 12% of the control group. Although ILR screening resulted in a three-times increase in AF detection and anticoagulation initiation, no significant reduction in the risk of stroke or systemic arterial embolism was noted. These findings might suggest that not all screen-detected AF merits anticoagulation^29,35^. Handheld ECG monitors such as MyDiagnostick or Merlin have also been studied; while these rely on automated algorithms and have sensitivities varying between 93% and 100%, detection rates of new AF have ranged from 0.9% to 7.4% only^26^.

While the aforementioned studies may suggest a potential role for population-wide AF screening, the modality of screening (short vs. long-term) and the characteristics of the screened population (including age) impacted the ability to use the tool and any subsequent detection of AF. The mean age of participants in the Huawei Study was 35 years, however, there was a mean age of 54 years in the suspected AF group^31^. In the Apple Watch study, only 5.9% of the total cohort were over 65 years of age^30^. These results suggest that smartphone/app/watch-based screening is likely to be of most value in older people given the high prevalence of AF in this population. However, lower technology literacy among older patients may lead to under-utilization of these tools leading to the continued presence of undiagnosed AF.

Rhythm tracings and PPG-enabled technologies do not confirm the diagnosis of AF, and subsequent ECG or continuous ECG recording is generally required for diagnosis. The variability in sensitivity and specificity of these monitoring modalities shown in the above trials raises concerns about false positive diagnoses leading to additional unnecessary tests, resulting in, psychological stress and additional costs^27^.

The 2020 ESC guidelines on AF screening have advised caution regarding the routine use of screening tools other than the standard of care ECG, especially given that current data on these tools were generated in observational cohorts, and no head-to-head comparisons have been reported^27^. The US Preventive Services Task Force (USPSTF) states that the current evidence is insufficient to assess the balance of benefits and harms of screening for AF^36^. Intermittent ECG recording ± pulse palpation can contribute to a nearly four-fold increase in new AF detection and provide immediate diagnosis, and it continues to be the gold standard when compared with the aforementioned screening methods^27^. Opportunistic, as opposed to routine, screening of individuals over the age of 65 years with additional stroke risk factors captured by CHA_2_DS_2_-VASc score appears to have the best evidence for new AF detection, while providing the best cost-effectiveness^37^. Analysis of the STROKESTOP study, with a 6.9-year follow-up, demonstrates the cost-effectiveness of population-based AF screening. Screening resulted in gained life years and quality-adjusted life years, with cost-saving demonstrated in 99.2% and 92.7% of simulations, emphasizing its viability, at least in elderly populations^38^.

It is unclear if treating incidentally diagnosed AF improves patient outcomes in general consumer populations. The ongoing HEARTLINE trial is attempting to answer this question^34^. In our opinion, at this time these technologies should be used only on an individualized basis, with a caution against overreliance.

### AF management

**Fig. 2:**
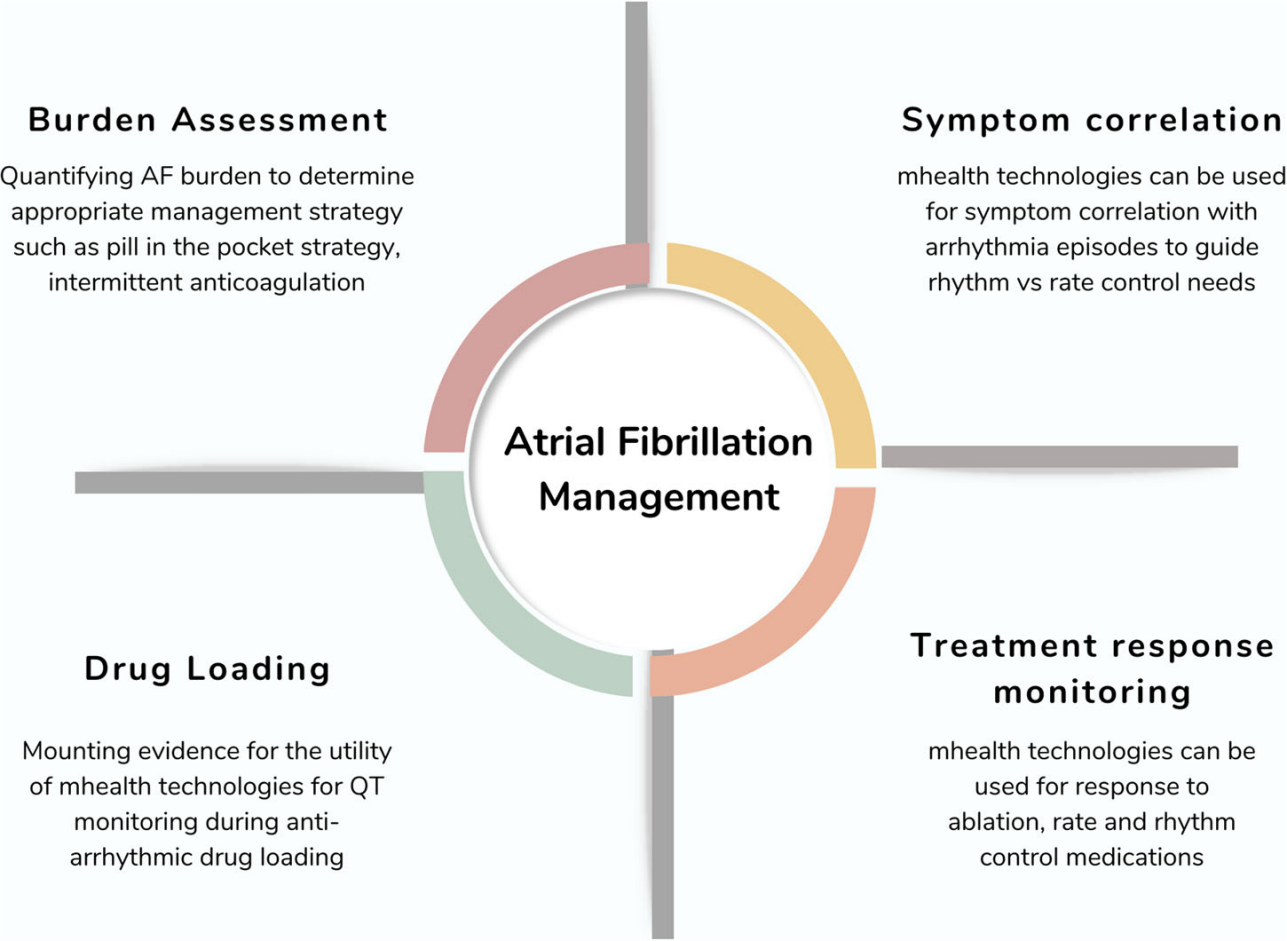
Incorporating mHealth technologies in AF management. Mobile technologies can aid the clinicians caring for AF patients in multiple ways including burden assessment, symptom correlation, treatment response monitoring, and drug loading. AF atrial fibrillation.

In most individuals, AF is a predictably recurrent disease. Indeed, paroxysmal AF recurrences follow a clustered pattern and persistent AF shows a significant time-dependent pattern of recurrence after rhythm control interventions fail^39^. Easily accessible, quickly scalable, and user-friendly mHealth technologies offer advantages over conventional tools and make them strong contenders for AF management (Fig. 2).

The clinical utility of these mobile technologies has largely been studied in groups with no prior known AF, directly influencing their predictive values. Validation of accuracy and predictability in those with known AF has not been done for these devices^40^. Indeed, the largest study of mobile ECG and AF, the Apple Heart Study, excluded patients with a reported diagnosis of AF, and its Food and Drug Administration (FDA) clearance states significant limitations during movement and when heart rate is below 50 bpm or above 150 bpm^30,41^.

Conceivably, mHealth technologies may help refine stroke risk in AF patients. Scoring systems consider the diagnosis of AF in a binary fashion (present or absent) rather than AF burden. Evidence suggests persistent AF has a higher risk of stroke, HF, and cognitive impairment than paroxysmal AF^42^. Several studies have shown that the risk of stroke is temporally related to the recency of onset of AF^43,44^. However, others have shown that ischemic stroke was temporally discordant from AF episodes such that most patients with ischemic stroke did not have AF in the preceding days or weeks^45^. These studies were performed in patients with pacemakers and implantable cardioverter defibrillators (ICDs), who may have higher background rates of ischemic stroke, and have not been reproduced with wearable devices or in lower risk cohorts. Still, quantifying AF burden as a percentage of time in AF over the monitoring period, rather than the absolute number of events, maybe a better indicator of stroke risk and has given rise to the concept of intermittent anticoagulation. This notion was tested in the iCARE-AF, REACT-COM, and TACTIC-AF pilot studies and showed that it is a feasible strategy and may decrease the risk of bleeding in low-risk patients with paroxysmal AF^46^.

Some trials have validated the ability of PPG and ECG-based wearables to detect AF in patients with a prior history of AF, use of anti-arrhythmic drugs, prior cardioversion, and ablation^19^. The “pill in the pocket” strategy, which involves carrying medication and using it only when needed, is well-suited for integration with mobile technologies. The potential advantages include a confirmatory validation of symptoms prior to therapy, earlier administration of antiarrhythmic drugs, closer monitoring, and confirmation of therapy success potentially avoiding emergency medical visits and side effects.

Additionally, QT monitoring is feasible with these devices, especially in patients receiving sotalol or dofetilide for rhythm control, as well as monitoring for recurrence after AF ablation^47^. A reliable indicator of recurrence is of the utmost importance as it has been well described that the perception of AF symptoms changes after ablation and recurrence is an AF ablation quality indicator^48^. Obtaining real-time reliable information will help clinicians make informed recommendations when treating patients.

The Heart Rhythm Society and European Heart Rhythm Association publications have offered practical advice concerning the use of wearables by patients for managing cardiovascular health and arrhythmias in various clinical situations. These papers outline tangible pathways for AF screening and management using digital technologies to enhance patient care^49–51^. Future research efforts will incorporate health apps and AI into treatment protocols as it has already been shown that the use of convolutional neural networks in the detection of AF is feasible^52^.

### Role of mHealth technologies in other arrhythmias

#### SVT

The paroxysmal nature of SVT and its unpredictability makes it a challenging arrhythmia to diagnose, as the arrhythmia often self-terminates before arrival at a healthcare facility for standard ECG recording. The diagnostic yield for traditional monitoring varies from as low as 10% for 24-h Holter monitors to 50–60% for medical-grade event monitors, illustrating that increased duration of monitoring improves yield. mHealth devices (ECG or PPG-based), facilitate relatively inexpensive, long-term rhythm monitoring and may successfully diagnose patients with brief episodes of sustained palpitations^53,54^. The resolution of smartphone-based single-lead ECGs is sufficient to differentiate SVT from a common misdiagnosis of sinus tachycardia for experienced ECG readers (89% sensitivity and 91% specificity) and to determine mechanism^55,56^. Despite this promise, only 51% of surveyed physicians indicated that they would proceed with an invasive EP study on the basis of a patient-recorded symptomatic, regular tachycardia via a handheld single-lead ECG system, illustrating the need for definitive prospective randomized trials^57^.

#### PVCs

PVCs are a common cause of palpitations and due to their irregular rhythm, mHealth devices may misdiagnose these as AF. Discrimination algorithms under development may resolve this issue. For example, in PULSE-SMART, a smartphone-based arrhythmia discrimination algorithm reliably discriminated PVCs from sinus rhythm, PACs, and AF with a 96% accuracy^58,59^. In another study, a simple computational algorithm filtered and extracted QRS features from an ECG device connected to a smartphone to construct a feature matrix. The algorithm, developed using the MIT-BIH arrhythmia database and clinically validated in a separate cohort of 100 participants, documented a PVC recognition accuracy of 98.69%^60^. While diagnosing and differentiating PVC is improved, quantification of burden may still need to be addressed.

#### VT

While the evidence supporting the use of smartphones for diagnosing ventricular arrhythmias is limited to case reports, these reports do suggest that this approach could be beneficial. In one case, non-sustained VT correlated with symptoms with exertional pre-syncope, leading to an EP study with induction of sustained right ventricular outflow tract VT and successful ablation^61^. In another case, recurrent syncope was associated with a recording of monomorphic VT on Apple Watch leading to a secondary prevention ICD placement^62^.

However, it is important to recognize that the practical application of wearables for VT diagnosis and management in the near future is less likely. While the widespread availability of smartphones allows patients to record SVT, PVCs, and VT, with subsequent accurate physician interpretation; the complexities and potential risks associated with VT necessitate a cautious approach. As VT diagnosis and management often require immediate intervention and specialized medical attention, the incorporation of wearable technology must be carefully evaluated and integrated into a broader clinical context. Clinical trials, such as the multi-center randomized control trial investigation of palpitations in the ED (IPED), may offer guidance on how best to incorporate this rapidly developing technology into clinical practice^63^.

### Role of mHealth in risk-stratification, prevention, and treatment of sudden cardiac death (SCD)

**Fig. 3:**
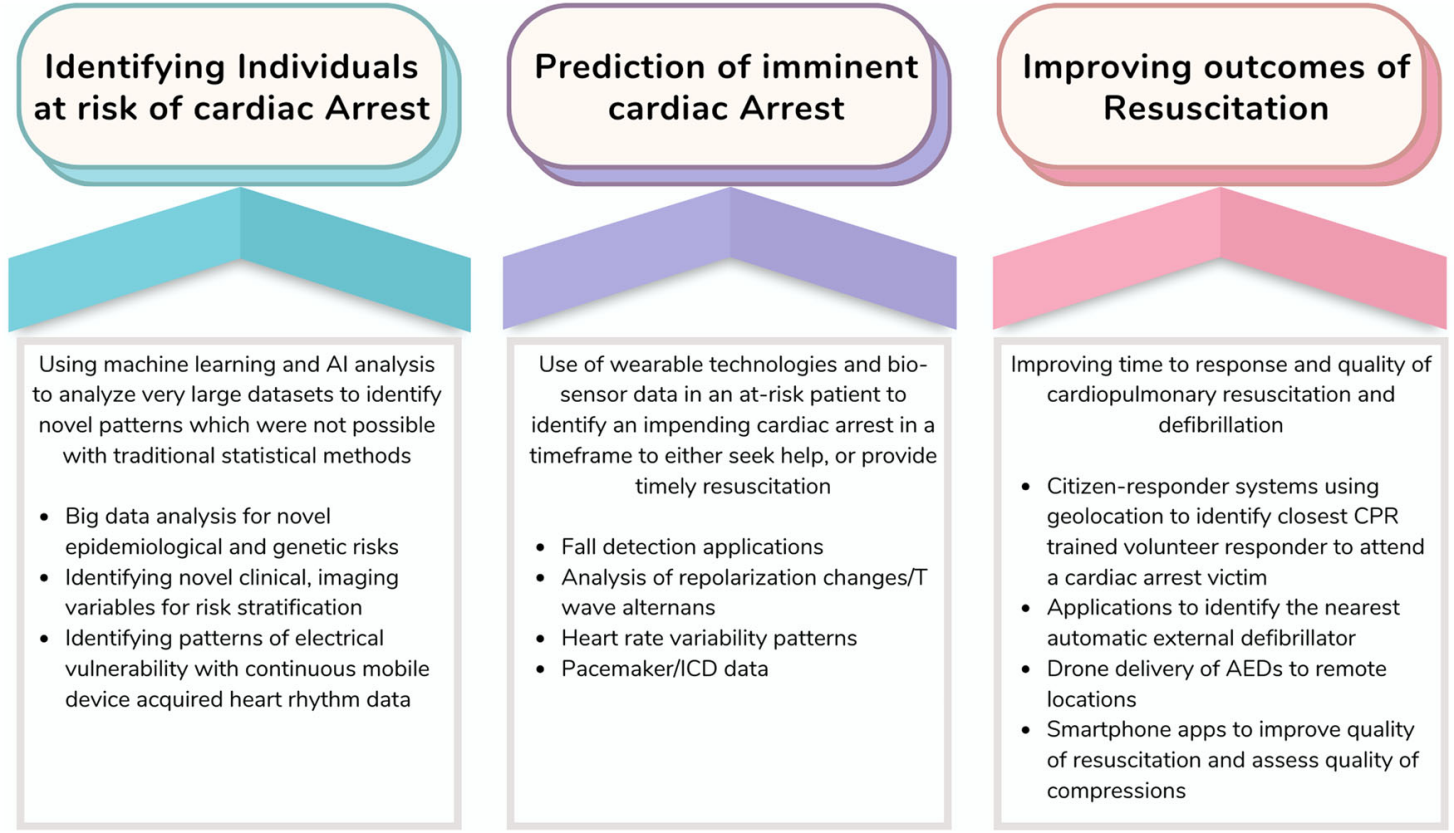
Role of mHealth technologies in the prevention and management of sudden cardiac arrest. This figure illustrates the various ways in which mHealth technology can be utilized in the prevention and management of sudden cardiac arrest. It showcases the use of ML and AI analysis to identify novel patterns, the application of wearable technologies and bio-sensor data for early detection, and the enhancement of response time and quality of resuscitation through innovative methods such as citizen-responder systems and drone delivery of AEDs. ICD implantable cardioverter defibrillator, CPR cardiopulmonary resuscitation, AED automated external defibrillator.

The implementation of mHealth and related technologies could affect the risk assessment and treatment of SCD in three ways: identifying individuals who could benefit from an ICD or other targeted intervention due to their high long-term risk and potential benefits; detecting individuals who may be at imminent risk of cardiac arrest; and reducing the time to defibrillation and/or enhancing the quality of resuscitation to improve the chances of survival^64^ (Fig. 3).

#### Identification of individuals at risk of SCD

While ICDs save lives in patients with clinical characteristics known to indicate a high risk of SCD, their impact on decreasing overall sudden death is low, as the large majority of sudden deaths occur in individuals without these indicators^65,66^. Better tools for risk prediction, particularly in the general population without known heart disease, are thus critical to decreasing SCA. AI and ML may address this critical need. In one example with immediate clinical relevance, ML has been used to create algorithms that can identify decreased LV function, known to predict ICD benefit, from the standard 12-lead ECG^67^.

Other ML algorithms have used complex computing to identify new variables that may predict the risk of SCA or overall mortality, whether from clinical or ECG patterns of variables^68,69^. Many factors predict long-term mortality in the general population. These include measures based on heart rate time series, such as heart rate variability (HRV) or heart rate recovery on stress tests, or nonlinear factors, which probably identify overall physiological stress, transduced through the autonomic nervous system or other processes^70,71^. Others identify vulnerable electrophysiological substrates, such as T wave alternans or QRST-integral. There are challenges in moving from prediction to improvement in mortality, for both these prior markers, and newer AI-based markers. To improve mortality, the marker must provide adequate positive and negative predictive value to drive a preventative intervention^72^. Transitioning from predicting mortality to improving it, is challenging for both traditional markers and newer AI-based markers. For a marker to effectively enhance mortality, it must possess sufficient positive and negative predictive values to support a preventive intervention. Alternatively, the marker should pinpoint modifiable physiological processes. While AI holds promise in overcoming these challenges, its effectiveness remains unproven.

Commercial companies advertise that wearable devices measuring HRV can be used as a tool to improve overall health. Limited data in controlled settings showed that HRV monitoring can optimize and avoid overtraining in athletes^73^. A study of the clinical relevance of diverse parameters captured via a wearable cardioverter-defibrillator (WCD) in recently diagnosed HF patients notably revealed that alterations in heart rate, step count, and HRV observed during WCD usage could be used as prognostic predictors for improvements in left ventricular ejection fraction (LVEF)^74^. However, currently, the role of HRV as a mHealth tool in the general population is untested in systematic clinical trials.

#### Prediction and identification of imminent cardiac arrest

Wearable watches can identify a fall and connect the wearer to emergency services^75^. Whether sensor-based biometric data, perhaps combined with fall identification and verbal feedback, could provide identification of an imminent SCA, is intriguing. In one early Holter-based study, dynamic changes in repolarization were seen in a 10-min time frame preceding ventricular arrhythmia^76^. The concept of a wearable device that can truly provide a warning signal of impending arrest is exciting, but far in the future^77^.

#### Improving the time to resuscitation

Improving the time to the performance of cardiopulmonary resuscitation (CPR), and defibrillation, is the most important, and modifiable, contributor to survival from SCA, and is perhaps the most important use of mobile technology for SCA to date. Since its first report in 2007, citizen-responder systems have not only increased layperson CPR, but also have improved survival to hospital discharge by 50%^78,79^. In these systems, citizens willing to provide assistance register in a first-responder network and those closest to a SCA patient based on geolocation are notified by text. Similarly, the use of mobile apps to identify the nearest automatic external defibrillator decreases the time to access the AED and may improve outcomes^80^. ML may further facilitate the use of drones for faster delivery of AEDs, although data on these systems to date are based only on simulations^81^. In addition to decreasing the time for CPR and defibrillation, smartphone apps may also improve the quality of resuscitation. In one simulation study, smartphone video-based analysis of CPR performance improved the quality of compressions^82^. The American Heart Association has recognized the importance of further research in leveraging digital strategies to improve survival from cardiac arrest^83,84^.

### Role of mobile technologies in arrhythmia care in children

While mHealth technologies are developed and validated in the adult population, there is rapid uptake of these tools in the pediatric and congenital heart populations. Studies show 53% of children have a tablet by age 11 and 84% own a smartphone at age 13^85,86^. Though the use of wearables in this group is poorly defined, their technological savvy may enable quick adoption.

A few mHealth technologies have been tested and validated compared to standard 12-lead ECGs in children, including the Alive Cor Kardia Monitor and Apple Watch for symptom-rhythm correlation and QT measurement with good patient/provider usability and data quality^55,87–89^. Automated algorithmic interpretation has been limited often requiring physicians over-read to ensure accuracy^87,88^. This should not be surprising given the training datasets used for algorithm development are from older adults with slower baseline heart rates (HRs) and tachycardia rates in contrast to the higher resting baseline HRs and tachycardia rates seen in younger patients. Patch-based wearable devices were more user-friendly than Holter and often provided high-quality data but may be limited by small chest surface area and patient intolerance to adhesive^90^.

### Use of mHealth application for care of non-arrhythmia conditions

mHealth applications have improved the care of patients with other cardiac conditions, which may have direct implications on atrial and ventricular arrhythmia development and disease course in these patients. We therefore highlight a few advances below.

#### Coronary artery disease

Various smart device-based mobile ECG systems have been studied for earlier detection of myocardial infarction (MI). In a study of multichannel ECG using a smartwatch, the sensitivity, and specificity for the diagnosis of ST elevation MI and non-ST elevation MI was greater than 90%^91^. Similarly, an FDA-approved ICM with an IS-1 lead implanted into the right ventricular apex for real-time monitoring of ST segment changes, allowed for early detection with 55% of patients presenting within 2 h of the MI^92^. Apart from early detection of MI, mHealth applications using smartphones and fitness trackers help improve adherence and cost-effectiveness of cardiac rehabilitation and also promote risk factor modification and compliance^93,94^. Larger studies are needed to validate the use of mHealth applications in both the early detection of MI and in increasing cardiac rehabilitation participation.

#### HF

mHealth could have a significant role in preventing HF hospitalization by recognizing HF exacerbations earlier based on vital signs and symptoms, thoracic impedance, and hemodynamic monitoring systems. In a meta-analysis of studies on mHealth interventions, there was a significant reduction in all-cause and CV mortality, as well as HF hospitalization^95^. Thoracic impedance monitoring with CRT and ICD devices improves HF management. The remote dielectric sensing system detects fluid overload in the lungs and thereby provides objective measurement of pulmonary edema leading to early therapy, preventing HF hospitalization^96^. Algorithms such as Heartlogic® identify early HF exacerbations by monitoring multiple parameters such as heart sounds, rate, and activity plus thoracic impedance, respiratory rate, and tidal volume. ALLEVIATE-HF is an ongoing study utilizing an ICM-based algorithm to reduce HF events. Hemodynamic monitoring with a PA pressure monitor (eg. CardioMEMS) has shown a significant reduction in HF hospitalizations by 37% over 15 months. Supplementary Table 1 outlines the relevant clinical trials on mHealth in HF.

#### Hypertension

Multiple studies have shown that mHealth platforms that allow tracking of BP by clinicians helped lower systolic and diastolic BP^97^. Currently, research studies are underway to show the feasibility and validity of PPG-based home BP assessment through wearables for continuous ambulatory checks leading to improved personalized hypertension management^98^.

#### DM

DM was one of the first diseases where mHealth-based management was successfully explored. Various mHealth platforms that encourage self-monitoring of blood glucose monitoring, medication, and diet compliance have been successful (0.3–0.4% reduction in HbA1C) enough to be recommended by international societies. Continuous blood glucose monitoring, and insulin delivery through smart pens and automated pumps have positively impacted DM management^99^. A full discussion of the transformative roles of mHealth and digital health technologies in diabetes management is beyond the scope of this article, but a comprehensive review on the subject has recently been published^100^.

#### Sleep apnea

Various mHealth applications have been used for diagnosis and follow-up of sleep apnea (OSA). Sleep trackers such as smart watches, wearable rings, smart mattresses, and other smartphone-based sensors can be used to screen for OSA. Similarly, newer CPAP machines allow better integration with mHealth allowing for tracking of CPAP use and helping improve compliance^101^.

### mHealth applications in clinical trials

mHealth devices are revolutionizing clinical trials by enabling a site-less approach, where participants can join and provide data remotely, without needing to visit a trial site^102^. These devices use Internet of Things (IoT) technology to actively and passively collect a broad range of health data. This method not only reduces the need for in-person visits but also expands trial participation to include people who are typically underrepresented, such as those with mobility-limiting disabilities or those living in remote areas with limited access to research centers^102,103^. Outside of cardiology, mHealth devices using frameworks such as Apple’s open-source Research Kit (http://researchkit.org/) have utilized smart device sensor data to study a variety of topics including increasing physical activity, rheumatoid arthritis symptom management, Parkinson's disease progression, seizure detection in epilepsy, and geriatric fall prevention^104–108^.

Before the advent of mHealth technologies, ambulatory ECG monitoring had already been widely utilized as a data collection modality in clinical trials. For example, the Catheter Ablation vs Antiarrhythmic Drug Therapy for AF (CABANA) trial employed a proprietary telephonic transmission two-lead ECG mobile cardiac telemetry device that facilitated patient-activated monitoring^109,110^. The Apple Heart Trial and Huawei Heart Study demonstrated the feasibility of PPG smart watch-based screening for AF^30,31^.

Traditional rhythm monitoring tools employed as gold standards in clinical trials are limited by the need for resource-intensive mobile cardiac telemetry and the time lag between data collection and interpretation in the case of patch monitors. These limitations can be overcome by mHealth for QT interval monitoring, which is an important consideration in pharmaceutical trials. Many potential therapeutics could prolong the QT interval, potentially resulting in increased risk for torsades de pointes (TdP)^111^. These trials require real-time monitoring so that investigators can assess the risk of QT prolongation and intervene in a timely manner to prevent TdP. A recent remote randomized trial of the known QT-prolonging agent's hydroxychloroquine and azithromycin for the treatment of COVID-19 utilized a six-lead KardiaMobile 6 L for ECG collection, allowing for accurate and timely ascertainment of the QTc, core laboratory validation and notification of results to the patient and treating physician^102,112^.

**Fig. 4:**
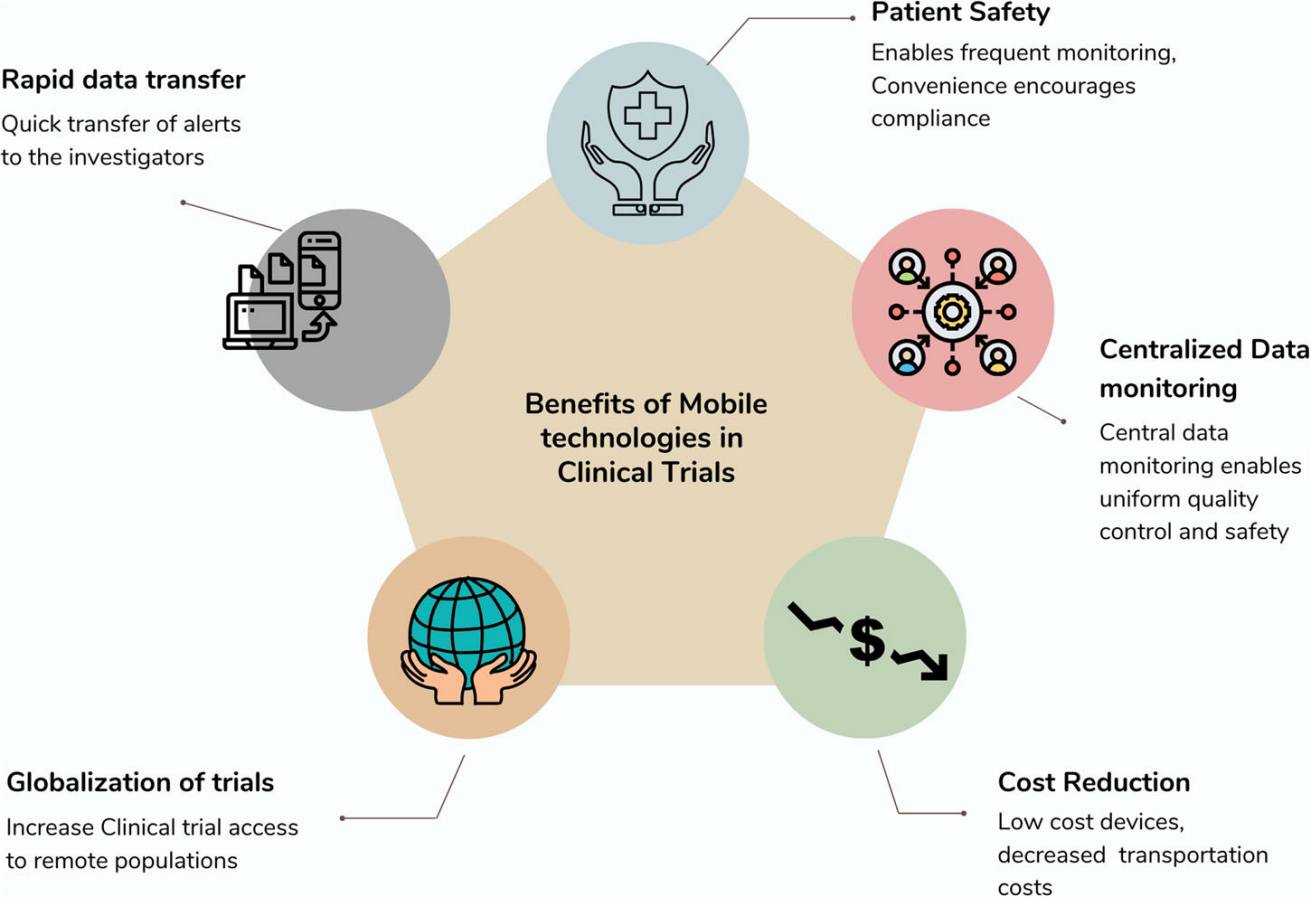
Enhancing clinical trials with mobile technologies. Integrating mobile technologies in clinical trials offers a range of benefits, from enabling frequent monitoring to increasing access for remote populations. These technologies facilitate quick data transfer, enhance patient safety through rapid alert notifications, and reduce costs associated with transportation and device expenses. Additionally, centralized data monitoring ensures uniform quality control and supports the globalization of clinical trials.

There are several potential advantages to using mHealth in cardiovascular research in general, and cardiac arrhythmia research in particular (Fig. 4). By digitizing trial elements, including enrollment, clinical history gathering, follow-up visits, and outcome assessment, the cost per participant may be reduced. In addition, this may allow broader representation by including participants who have travel impediments or are historically underrepresented in trials due to structural disparities^30^. While this approach has the potential to enhance the diversity and inclusivity of participants, it comes with an inherent risk of disadvantaging those with limited means and lower technological literacy^113^. Careful attention must be paid to diversity and equity as the adoption of mHealth tools for clinical research continues to grow^26^. Further, as highlighted by a 50% attrition rate in completion of the Apple Heart virtual study visit, future studies relying on mHealth will need to account for potentially increased loss to follow-up^30^.

## Limitations and challenges caused by digital health technologies

**Fig. 5:**
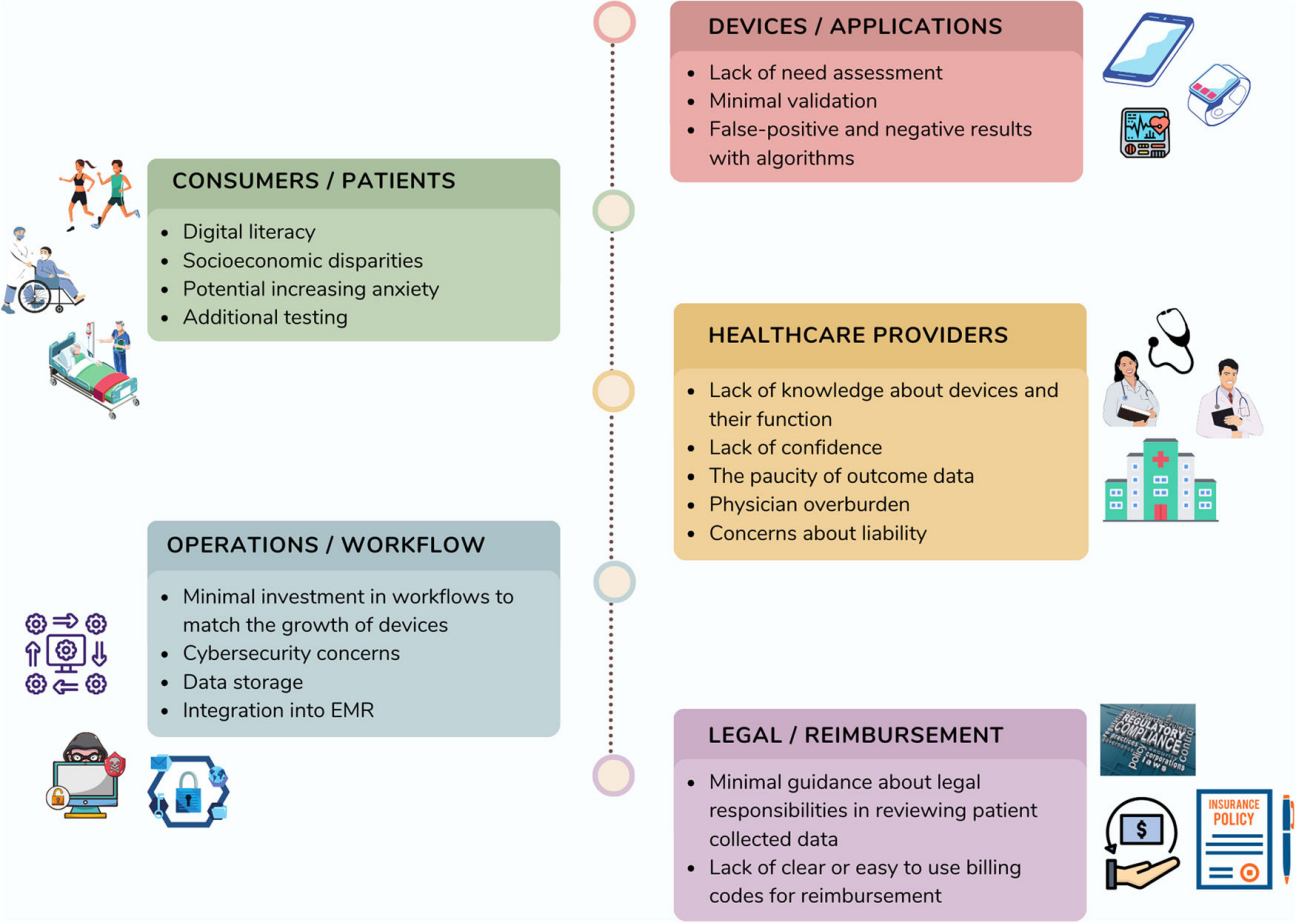
Limitations and barriers to wide clinical adoption of digital health technologies. The integration of digital health technologies in the care of arrhythmia patients presents a myriad of challenges across different stakeholders. Healthcare providers face hurdles such as a lack of understanding of device functionalities, limited confidence in their use, and concerns about liability issues. For consumers and patients, challenges include varying levels of digital literacy, socioeconomic disparities affecting access, potential anxiety from test results, and the need for additional testing. Operational challenges encompass inadequate investment in workflows to accommodate the increasing number of devices, cybersecurity risks, data storage issues, and the integration of device data into electronic medical records. Moreover, the lack of clear guidance on legal responsibilities and reimbursement processes further complicates the adoption of these technologies in the healthcare landscape. EMR electronic medical record.

Despite the exponential growth in direct-to-consumer devices and the large investments in digital technologies, many challenges remain and limit the wider adoption of these digital technologies in clinical practice (Fig. 5). Wearables and apps directed toward healthcare often lack basic needs assessment and many are released to the market with little if any validation^26,114^. Some are paired with automated algorithms that provide instant interpretation and feedback to the patient. Rhythm interpretation is an example of such algorithms and in general, whilst good they are far from perfect^115,116^. Review by a clinician is still needed before any clinical decision is made^117,118^. The magnitude of false-negative results and the clinical implication of detection of asymptomatic AF remain uncertain.

Digital literacy and socioeconomic status remain important obstacles. While smart devices are widely available, their adoption follows consumer base patterns. Based on consumer research, one in five Americans use a smartwatch or fitness tracker, with greater concentration in persons between 18 years old and 49 years old, having higher levels of income and education, creating a disparity in the delivery of care^119^. Many older patients do not use the smart features and need help in downloading and initiating an app or a wearable device. Such support is not always available in clinics where resources are limited, and family assistance may not always be present. While consumer devices are less expensive than medical devices, patients may still face cost-related challenges since most health insurance providers do not provide coverage for them. This leads to a bias towards the young, privileged, and generally healthier population who are less likely to need health monitoring. However, many studies have shown that compliance is not related to age, with older patients being more compliant than their younger counterparts once they start using these devices^19,120^. Positive findings, both true and false, could raise the anxiety level of patients and can lead to more testing and healthcare costs^26,35^.

When patients or consumers are faced with abnormal findings from these devices, they might not have a skilled clinician able or willing to interpret the data in a timely manner, for various reasons. Clinicians might lack technical knowledge about the devices, and many are concerned about being expected to provide technical support to their patients. Some clinicians might lack the expertise in interpreting the findings, or simply lack the clinical guidance regarding the proper response to a detected abnormal rhythm, such as randomly detected AF in an otherwise healthy individual^29,35^. Some clinicians fear the deluge of data that often leads to a time lag between actionable events and the ability to provide the proper over-read of the findings or intervention, leading to more clinician burnout^121^.

Innovation in wearables and apps has not occurred in parallel with the required advances in platforms that would enable clinicians to scale the use of these technologies to all patients. E-mails and messages with attachments pose a safety and security risk when a proper workflow to properly triage, manage, or store the data is absent^19,114^. Some innovative cloud-based platforms have been successfully implemented to monitor patients with AF^122^. However, even when such platforms exist, they lack interoperability to upload ECG data from other wearables or different operating systems. In addition, the data need to be incorporated into the electronic medical record (EMR) of the patient, which is often not possible^19,122^.

For mHealth to flourish in cardiology, the conventional model in which care processes are grounded in physical visits to healthcare professionals must be reimagined. New care processes that seamlessly integrate with mHealth technologies are required. This transformation would require the establishment of call centers and a hub of tele-consultations (perhaps operated by telehealth nurses) that offer patients a new way to access healthcare expertise. Finally, without legal clarity about the expectations and the responsibility of reviewing patient-collected data in a timely fashion, in addition to the lack of clear and easy-to-use reimbursement models, wide adoption and scaling of using wearables in clinical practice will be limited and could improve medicine for the few rather than impact the health of the general public^123^.

### Regulatory issues and reimbursement challenges

#### Regulatory issues

**Fig. 6:**
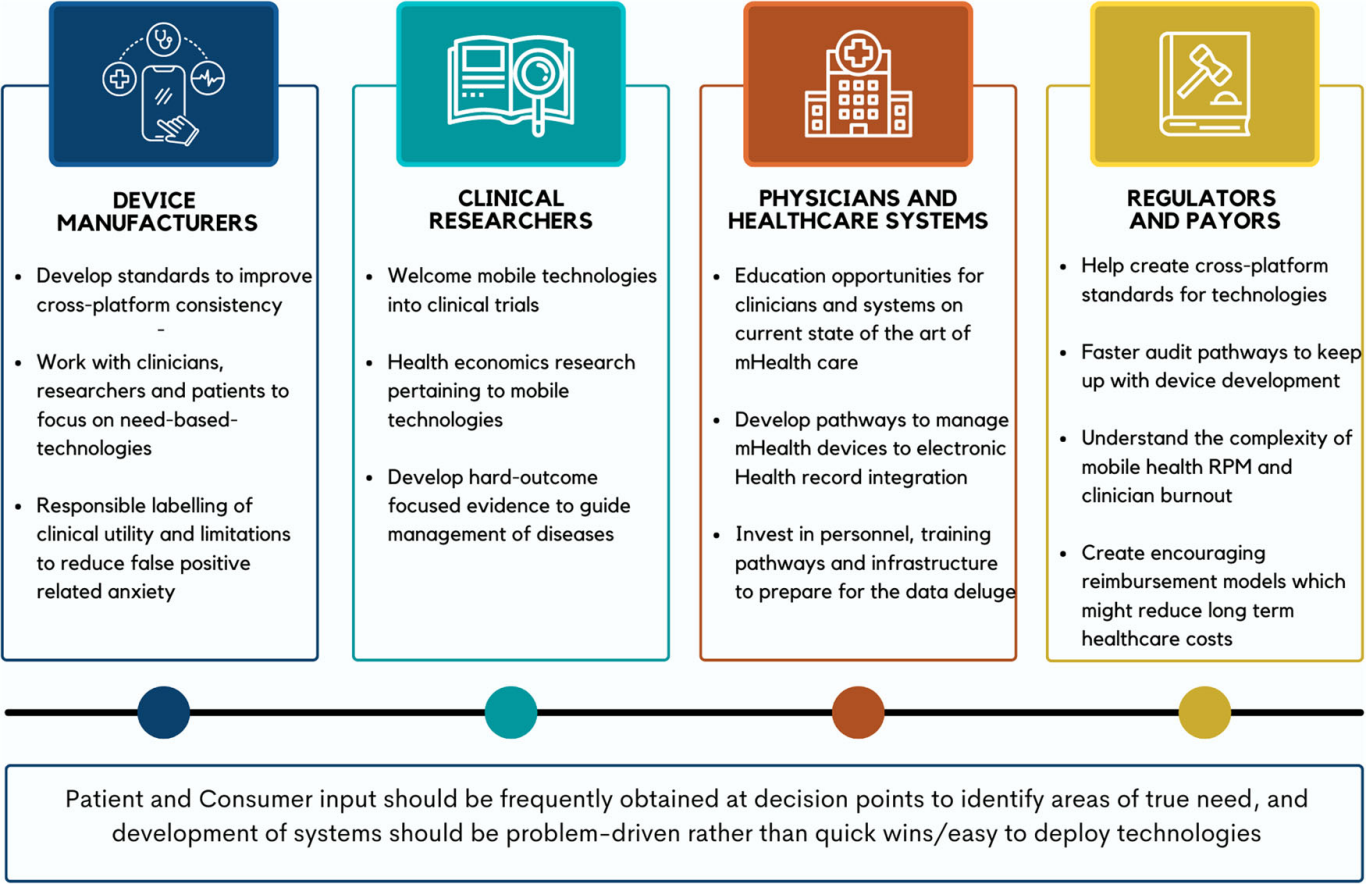
Future of mHealth driven arrhythmia care. Innovation and development considerations for the different stakeholders. The figure illustrates a comprehensive roadmap for advancing mHealth technology in arrhythmia care through collaboration among different stakeholders. It emphasizes the importance of setting standards for cross-platform consistency, engaging with clinicians, researchers, and patients to address specific technological needs, providing clear labeling of clinical utility, welcoming mobile technologies in clinical trials, and investing in education and infrastructure to support data integration and management. The roadmap also highlights the significance of developing evidence-based strategies, ensuring regulatory compliance, and involving patients in decision-making processes to drive innovation in arrhythmia care. RPM remote patient monitoring.

The United States FDA is expanding staffing, digital health policy, and guidance in a dynamic process, trying to keep pace with device developers. The FDA Digital Health Center for Excellence (DHCE) has a goal to empower stakeholders with objectives to connect and build partnerships, share knowledge, and innovate regulatory approaches^124^. DHCE is establishing new programs and pathways to promote more efficient review and approval of digital health devices and software, including those with AI and ML (Fig. 6). New FDA software as a medical device (SaMD) programs include software pre-certification, predetermined change control, good machine learning practices (GMLP), and real-world performance (RWP) monitoring^125^. New FDA regulatory pathway programs including the breakthrough devices program and safer technologies program promote the development and efficient FDA review of new technologies^126^. Arrhythmia care is highly represented in the first wave of digital health devices thus industry and stakeholders can help steer FDA regulatory pathways and requirements by participating in interactive workshops.

Historically, the US requirements for medical devices have been considered more rigorous than in the EU as they are supported with more detailed regulations and guidance, with more FDA staff to review submissions^127^. The adoption of the regulation (EU) 2017/745 following a four-year transition period has changed the landscape. While EU MDR is not specific regarding digital health and software, the EU adoptions of IMDRF/SaMD WG/N12 FINAL: 2014 and 2019 guidance medical device software (MDSW) shift definitions, classification, risk assessment, and conformity to be similar to those in the US, elevating most MDSW to class IIa or class III^128^.

Under the current regulatory frameworks in the US and EU, there is potential for a more unified approach to the development and approval of digital health technologies and SaMD. By comparing and aligning the regulatory requirements, including software validation protocols, developers may identify a single streamlined approach that can be used for both US and EU markets.

However, it is essential to also consider the regulatory landscapes in other significant markets, such as Asia, Africa, and Latin America. Countries in these regions are rapidly adopting digital health technologies and have unique regulatory requirements that must be addressed. For instance, Japan has established a clear regulatory pathway for digital health and SaMD devices under its Pharmaceuticals and Medical Devices Agency (PMDA)^129^. South Korea’s Ministry of Food and Drug Safety (MFDS) has also implemented comprehensive guidelines for the approval of AI-based medical devices^130^. In India, the Central Drugs Standard Control Organization (CDSCO) is progressively developing regulations to manage digital health and SaMD products^131^. Similarly, Brazil’s National Health Surveillance Agency (ANVISA) has been updating its regulatory framework to keep pace with innovations in digital health technologies^132^. Aligning regulatory strategies across these diverse markets can facilitate broader global adoption and access to innovative health technologies.

The development of technology, including AI and ML, will continue to push regulatory boundaries. US and EU regulatory bodies, along with those in other key regions, must evolve to ensure that new devices are brought to market safely and efficiently. Industry support for initiatives by the FDA, EU, and other international regulatory bodies is crucial to this effort, promoting the global harmonization of standards and ensuring that innovative technologies reach the public without unnecessary delays.

#### Reimbursement issues

A critical link to continued development and availability of digital health solutions is having viable pathways to monetization, for developers and healthcare providers. Device developers can price their products in a variety of ways (flat fee, leasing fee, and service fee) but potential users must be able to reimburse both access to devices and software, and their time undertaking remote visits and reviewing an extensive amount of patient-generated data. Navigating the process of securing reimbursement from payers, including insurance companies and government programs, can be challenging and complex.

Remote patient monitoring (RPM) is often done voluntarily with costs absorbed by overhead or related charge codes when data is reviewed in coordination with remote or in-person visits. Larger-scale reimbursement solutions are required as remote monitoring becomes more frequent and more extensive in scope. The COVID-19 epidemic accelerated a trend that is very likely to continue.

In Germany, “DiGA” (Digitale Gesundheitsanwendung) represents a subset of digital health solutions vetted by the Federal Institute for Drugs and Medical Devices (BfArM)^133^. DiGAs, qualified for reimbursement by statutory health insurers, undergo rigorous BfArM evaluation for efficacy, safety, and data privacy. Once approved, they can be prescribed by clinicians and are subject to potential patient co-payments based on insurance specifics. The implementation of DiGAs exemplifies Germany’s strategy to assimilate digital health innovations into its healthcare infrastructure, facilitating a balance between innovation and regulatory oversight. Similarly, other European Union countries have developed regulatory and reimbursement models to evaluate and promote the development of digital health solutions. The European Commission has also been active in promoting a Digital Single Market, which includes initiatives to harmonize and promote digital health across member states.

Ultimately, a comprehensive review and expansion of digital health reimbursement policy and procedures is needed (Fig. 6). Procedures should be modular and flexible to accommodate the many different types of services associated with RPM (data frequency, complexity, multiple reviews, etc.). In addition to fee-for-service, a forward-thinking model could include incentives for value-based milestones associated with RPM such as lower number of office visits, fewer adverse events, earlier diagnoses, and no escalation of conditions. Such incentives would lead to improved care for patients and lower overall costs for payers by leveraging mHealth.

### Innovation and areas for future research

There has been extraordinary progress in the design, testing, and validation of consumer-facing cardiovascular technologies. Three irregular pulse detection AF studies collectively enrolled over one million participants^30–32^. Consumers have recorded over 150 million smartphone-connected ECGS (David Albert, 2022, 150 Million consumer smartphone-connect ECGs, personal communication). There is greater societal and consumer awareness of AF and other forms of silent heart disease.

One major problem is the need for seamless integration of wearable device-generated data into EMRs which can be condensed appropriately to highlight relevant information. Even with actionable data, clinical review and decision-making are necessary, which are again gated by the most precious resource in health care, clinician time. Semi-automated and automated systems are needed to manage diseases effectively. For example, algorithms—whether rule-based, AI-guided, or a combination of both—can empower patients to manage their own medical therapy according to established guidelines for conditions such as hypertension, DM, or HF. These systems can reduce the need for frequent clinician intervention and office visits, while also aiding in maintaining consistent and optimal treatment. This could also be done in AF to identify when to increase AV nodal agents for rate control, and when to escalate rhythm control (including catheter ablation). While these systems should start in a semi-automated fashion, there could be potential to move toward progressive automation.

Ultimately, clinical evidence is needed not only to validate whether a device can measure its intended physiologic data, but also to determine whether the intervention driven by the detected findings improves outcomes. Several studies are now underway to help answer this question. The HEARTLINE trial aims to enroll 25,000 patients to determine whether a strategy of wearable-guided AF screening, coupled with downstream anticoagulant adherence, can improve clinical outcomes of stroke, cardiovascular hospitalization, and death^34^.

## Concluding remarks

Mobile devices have the potential to revolutionize cardiovascular care, offering enhanced collaboration between doctors and patients and granting individuals agency in managing their health. These innovations introduce fresh strategies for disease screening, diagnosis, and management, potentially leading to better outcomes, streamlined healthcare, and cost savings. These innovations introduce fresh strategies for disease screening, diagnosis, and management, potentially leading to better outcomes, streamlined healthcare, and cost savings. Nevertheless, challenges encompass concerns about reliability, health data privacy vulnerabilities, and the potential to widen health disparities. Effective integration of these technologies necessitates collaboration and ongoing research to tailor them effectively to the needs of consumers, patients, and healthcare providers.

## Supplementary information

Supplementary Information
