# Supplementary material for: State of the art of mobile health technologies use in clinical arrhythmia care
Source: Commun Med (Lond). 2024 Oct 29;4:218. doi: 10.1038/s43856-024-00618-4 (PMC11522556; doi:10.1038/s43856-024-00618-4)
Supplement: Supplementary file 1 — Supplementary Information [file 43856_2024_618_MOESM1_ESM.pdf]

Supplementary Table 1 – Clinical trials studying heart failure outcomes using digital health and mHealth based monitoring and interventions

Type of monitoring	Study	Sample size	Intervention	Outcomes
Vital sign and weight measurement	Tele-HF[1]	1653	Telephone based, Symptom and weight	No reduction in mortality or HF hospitalization
	TIM-HF[2]	710	Mobile based,<br>daily EKG, BP and weight	No reduction in mortality or HF hospitalization
	BEAT-HF[3]	1437	Telephone based,<br>Weight, BP, HR and symptoms	No reduction in mortality or HF hospitalization<br>Better QoL score at 180 days
	TIM-HF2[4]	1571	Web based,<br>Weight, BP, HR, SpO2, EKG and self-rated health status (scale of 1 to 5)	Reduction in all-cause mortality and loss of days due to CV admissions
Thoracic impedance and fluid monitoring	multiSENSE[5]	900	Device based ICD or CRT, Heart sounds, thoracic impedance, RR, tidal volume, HR, patient activity	70% sensitivity of heartlogic algorithm to detect HF event.<br>Median days before HF event – 34 days
	Opti-Link HF[6]	1002	Device based ICD or CRT, thoracic impedance	No reduction in mortality or HF hospitalization.<br>24% impedance alerts not transmitted
	DOT-HF[7]	335	Device based ICD or CRT, thoracic impedance	No reduction in mortality or HF hospitalization
	SMILE[8]	268	Device based, ReDS system Thoracic impedance	48% reduction in HF readmission
	LINK-HF[9]	100	Device based, Multisensor chest patch	76% sensitivity of algorithm to detect HF hospitalization.<br>Median days before HF admission – 6.5 to 8.5 days
	BMADHF[10]	500	Device based, µCor thoracic fluid monitoring patch	Ongoing. To evaluate mortality and HF hospitalization
Pulmonary artery pressure monitoring	CHAMPION[11]	550	Device based CardioMEMS, Pulmonary artery pressures	37% reduction in HF hospitalization
	GUIDE-HF[12]	1022	Device based CardioMEMS, Pulmonary artery pressures	No reduction in mortality or HF hospitalization. Pre-COVID 19 subgroup showed reduction in HF hospitalization.

References

1. Chaudhry, S.I., et al., *Telemonitoring in Patients with Heart Failure.* New England Journal of Medicine, 2010. **363**(24): p. 2301-2309.
2. Koehler, F., et al., *Impact of Remote Telemedical Management on Mortality and Hospitalizations in Ambulatory Patients With Chronic Heart Failure.* Circulation, 2011. **123**(17): p. 1873-1880.

3. Ong, M.K., et al., *Effectiveness of Remote Patient Monitoring After Discharge of Hospitalized Patients With Heart Failure: The Better Effectiveness After Transition–Heart Failure (BEAT-HF) Randomized Clinical Trial.* JAMA Internal Medicine, 2016. **176**(3): p. 310-318.
4. Koehler, F., et al., *Efficacy of telemedical interventional management in patients with heart failure (TIM-HF2): a randomised, controlled, parallel-group, unmasked trial.* The Lancet, 2018. **392**(10152): p. 1047-1057.
5. Boehmer, J.P., et al., *A Multisensor Algorithm Predicts Heart Failure Events in Patients With Implanted Devices.* JACC: Heart Failure, 2017. **5**(3): p. 216-225.
6. Böhm, M., et al., *Fluid status telemedicine alerts for heart failure: a randomized controlled trial.* Eur Heart J, 2016. **37**(41): p. 3154-3163.
7. Veldhuisen, D.J.v., et al., *Intrathoracic Impedance Monitoring, Audible Patient Alerts, and Outcome in Patients With Heart Failure.* Circulation, 2011. **124**(16): p. 1719-1726.
8. Abraham, W.T., et al., *Primary results of the Sensible Medical Innovations Lung Fluid Status Monitor allows reducing readmission rate of heart failure patients (SMILE) trial.* Journal of Cardiac Failure, 2019. **25**(11): p. 938.
9. Stehlik, J., et al., *Continuous Wearable Monitoring Analytics Predict Heart Failure Hospitalization.* Circulation: Heart Failure, 2020. **13**(3): p. e006513.
10. *https://clinicaltrials.gov/ct2/show/study/NCT03476187*. 12/4/2021]; Available from: https://clinicaltrials.gov/ct2/show/study/NCT03476187.
11. Abraham, W.T., et al., *Wireless pulmonary artery haemodynamic monitoring in chronic heart failure: a randomised controlled trial.* Lancet, 2011. **377**(9766): p. 658-66.
12. Lindenfeld, J., et al., *Haemodynamic-guided management of heart failure (GUIDE-HF): a randomised controlled trial.* The Lancet, 2021. **398**(10304): p. 991-1001.
